# *This* and *That* Revisited: A Social and Multimodal Approach to Spatial Demonstratives

**DOI:** 10.3389/fpsyg.2016.00222

**Published:** 2016-02-16

**Authors:** David Peeters, Aslı Özyürek

**Affiliations:** ^1^Neurobiology of Language Department, Max Planck Institute for PsycholinguisticsNijmegen, Netherlands; ^2^Centre for Language Studies, Radboud UniversityNijmegen, Netherlands

**Keywords:** referential communication, language, space, demonstratives, gesture, pointing

As humans, we have the capacity to refer to the things in the world around us. In everyday spoken communication, we often use words to describe intended referents (such as objects, people, and events), and our bodies (e.g., eyes, head, and hands) to indicate the location to which our addressee should focus her attention in order to further identify what we are talking about (Bühler, 1934; Clark and Bangerter, 2004). Traditionally, referring has been described as an autonomous and addressee-blind act that speakers do on their own without taking into account beliefs about their addressees' knowledge about a referent (e.g., Olson, 1970; see Clark and Bangerter, 2004). In contrast, more recent views consider it rather a collaborative enterprise that requires that speaker and addressee work together, for instance in reaching mutual agreement on how to conceptualize and name a particular entity (e.g., Clark and Wilkes-Gibbs, 1986; Brennan and Clark, 1996; Clark and Bangerter, 2004). Such agreement is established through interaction, and the addressee is at least as important as the speaker in reaching agreement and establishing reference.

In prototypical instances of successful referring, speakers often produce spatial demonstratives like *this* and *that* to establish joint attention between speaker and addressee to a visible entity (Bühler, 1934; Levinson, 1983). Such demonstratives are among the most frequently used words in language, among the first words infants produce (Clark and Sengul, 1978), and possibly primordial in phylogeny (Diessel, 2006; Tomasello, 2008). Surprisingly, despite the advances made toward a social, collaborative account of referring more generally, the prevailing theoretical view on spatial demonstratives has remained deeply individual and *egocentric*, as illustrated by the following claims:

1. “[T]he anchoring point of deictic expressions is egocentric (or, better, speaker-centric). Adult speakers skillfully relate what they are talking about to this me-here-now” (Levelt, 1989, p. 46).
2. Spatial demonstratives “indicate the relative distance of an object, location, or person vis-à-vis the deictic center (…), which is usually associated with the location of the speaker” (Diessel, 1999, p. 36).
3. “[D]emonstratives are interpreted based on the speaker's body” ((Diessel, 2014), p. 122).

This egocentric account is intuitively appealing and still influential (e.g., Diessel, 2014; Stevens and Zhang, 2014). In the current paper, we question this account from both the production and the comprehension side, and discuss recent accumulating observational, experimental, and neuroscientific evidence that suggests an alternative social and multimodal view of demonstrative reference.

## Production of demonstratives: beyond egocentricity and relative distance

Although it is generally acknowledged that demonstratives have a social function in establishing joint attention to a referent (e.g., Diessel, 2006), the egocentric account claims that when using a demonstrative “the speaker, by virtue of being the speaker, casts himself in the role of ego and relates everything to his viewpoint” (Lyons, 1977, p. 638). Diessel (2014, p. 128) even states that “speakers of all languages employ an egocentric coordinate system that is anchored by the speaker's body at the time of the utterance,” and argues that the speaker's body is a conventionalized aspect of the demonstrative's meaning (Diessel, 2014, p. 122).

But are speakers really egocentric when using a spatial demonstrative? Analyses of everyday multimodal and face-to-face spoken corpora suggest the opposite. Küntay and Özyürek (2006), for instance, show that speakers of Turkish use the demonstrative ş*u* specifically for referents that are not yet in the *addressee's* visual focus of attention and the demonstrative *o* for referents that are in the addressee's visual focus of attention (see also Özyürek, 1998). Thus, speakers would not use an egocentric coordinate system, but rather take the viewpoint of their addressee into account. Jungbluth (2003), furthermore, reports that the physical orientation of *both* interlocutors relative to each other in a conversation drives demonstrative choice in Spanish. When speaker and addressee are face-to-face in a conversational dyad, all referents within the dyad are treated as proximal “without any further differentiation” (Jungbluth, 2003, p. 19). Hence, when using a demonstrative, speakers may not be that egocentric after all.

Critically, the egocentric account generally claims that spatial demonstratives mainly express a distance contrast (e.g., Lyons, 1977; Anderson and Keenan, 1985; Diessel, 1999, 2006, 2014; Coventry et al., 2008). In the case of simple two-term demonstrative systems, this means that a proximal demonstrative (English *this*) indicates a referent relatively nearby the speaker and a distal demonstrative (English *that*) indicates a referent relatively remote from the speaker's location. For three-term systems it has been argued that the ‘medial’ demonstrative is used for entities close to the addressee or for entities at middle distance from the speaker. Diessel (2014, p. 123) claims that such “distance specifications of demonstratives are universals.” However, descriptions of demonstrative systems in terms of relative distance (either to speaker or addressee) are often based on linguistic intuitions and not on extensive analyses of everyday communication or rigorous experimental testing. Observational and experimental studies suggest that relative distance to the speaker is often not primarily driving a speaker's demonstrative choice.

Enfield (2003, p. 104), for instance, in describing the Lao two-term demonstrative system, concludes that “distance cannot be what distinguishes the meanings of these two demonstratives.” Rather, demonstrative reference is described as a social, interactive process in which the choice for a proximal or distal demonstrative depends on how interlocutors perceive and interpret the physical space during their interaction (Enfield, 2003). What is perceived as “proximal” may depend, for instance, on the engagement areas of speaker and addressee during their conversation (Enfield, 2003; see also Hanks, 1990). Piwek et al. (2008), moreover, argue that demonstrative choice in Dutch is not driven by the relative distance of a referent to the speaker, but by the cognitive and visual accessibility of a referent to speaker and addressee (see also Burenhult, 2003; Jarbou, 2010). Experimental studies supposedly showing effects of relative distance (Coventry et al., 2008, 2014) also show that what is considered as nearby or faraway is very flexible, for instance depending on whether participants point with their finger or with a stick, and on a referent's (context-dependent) visibility, familiarity, and ownership properties. This flexibility suggests that, rather than actual physical proximity, perceived (psychological) proximity is a more important factor in demonstrative choice (see below).

## Comprehension of demonstratives: beyond egocentricity and relative distance

Due to its focus on the speaker, the egocentric view of demonstrative reference generally does not consider how addressees comprehend the demonstratives they hear. However, according to Diessel (2014), demonstratives are interpreted (by an addressee) based on the relative distance of an entity to the speaker's body. In this view, an addressee will expect that a speaker uses a proximal demonstrative in reference to an entity that is relatively close to the speaker's body at the time of the utterance and a distal term for entities relatively farther away from the speaker. This claim is again purely based on linguistic intuitions and not on empirical testing.

Studies actually investigating demonstrative comprehension are scarce. Stevens and Zhang (2013, 2014) presented participants with visual scenes that included a speaker, a hearer, and a referent, while they listened to an auditory stimulus that contained a demonstrative (e.g., *this/that cat*) and while their electroencephalogram (EEG) was recorded. The referent was either near the speaker, near the hearer, or away from both, and participants were asked to judge whether the demonstrative matched the visual scene. Participants' linguistic judgments were in line with the egocentric view of demonstrative reference. However, analysis of their EEGs suggested that they took into account whether speaker and hearer both gazed at the referent or not (Stevens and Zhang, 2013) and whether the speaker produced a pointing gesture to the referent or not (Stevens and Zhang, 2014). Thus, a measure tapping into linguistic intuitions (the judgment task) was found to be in line with the egocentric view whereas a measure reflecting online processing (EEG) found an influence of social factors such as the presence of shared gaze.

**Figure 1:**
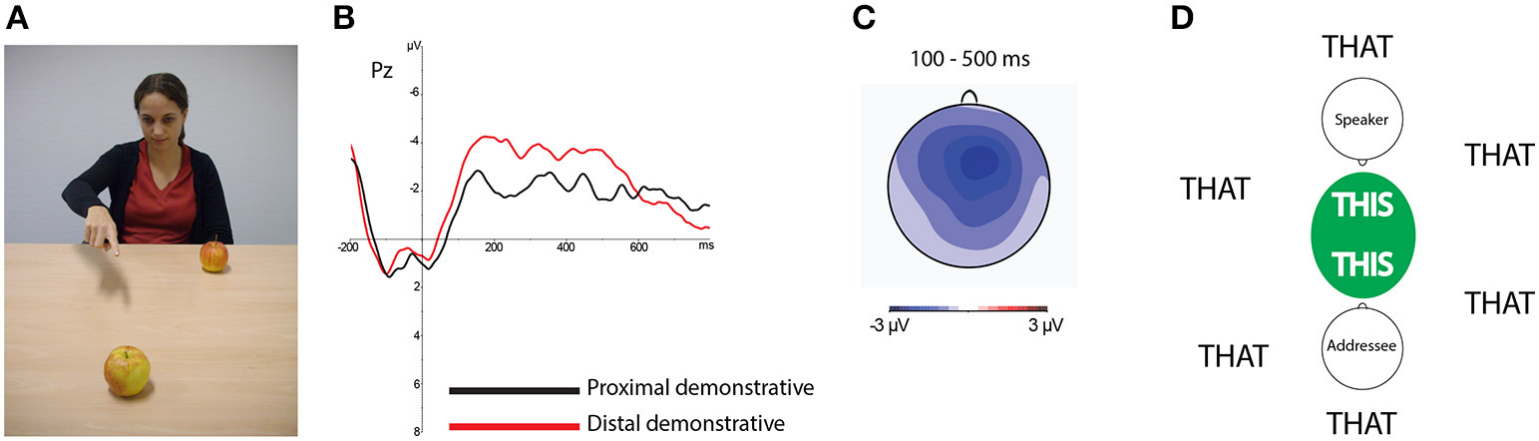
**(A)** Participants in Peeters et al. (2015b) were presented with picture stimuli in which a person pointed at one of two objects while they listened to an auditorily presented sentence that contained either a proximal or a distal demonstrative (e.g., “I have bought *this/that* apple at the market”). **(B)** Analysis of participants' event-related potentials (ERPs) as derived from their electroencephalograms (EEGs), time-locked to the onset of the demonstrative, suggested a higher processing cost for distal compared to proximal demonstratives when both objects were in the shared space between speaker and participant (as in the picture), irrespective of the distance of the referent-object to the speaker. **(C)** This effect had a fronto-central distribution over the scalp. The topographic plot shows the locus of the effect over the scalp averaged between 100 and 500 ms after the onset of the spoken demonstrative. **(D)** This finding suggests that speaker and addressee may create a shared space in which all referents become psychologically proximal.

Recently, Peeters et al. (2015b) investigated demonstrative comprehension in a paradigm in which participants listened to sentences that contained a demonstrative while they saw a picture of a speaker manually pointing at one of two visible objects. Higher processing costs were found for comprehending distal compared to proximal demonstratives when referents were in the shared space between speaker and participant (see Figure 1). Addressees thus took into account whether a referent was inside or outside the space that was shared with the speaker. No effect of the relative distance of the referent to the speaker was found. These findings suggest that demonstrative comprehension is sociocentric and involves the *we*-here-now (Peeters et al., 2015b), rather than egocentric and driven by the *me*-here-now (Levelt, 1989).

In sum, paradigms going beyond simple intuitions show that demonstrative reference, from both a production and a comprehension perspective, is a joint action rather than an egocentric, addressee-blind phenomenon.

## A social and multimodal approach to demonstrative reference

The findings discussed above seriously question the egocentric view that demonstratives express a distance contrast as calculated from the speaker's location. We propose a social alternative: Demonstrative production and comprehension are not primarily governed by the physical proximity of a referent to the speaker, but rather by the *psychological* proximity of a referent to *both* speaker and addressee. Moving beyond other social accounts (e.g., Enfield, 2003; Jarbou, 2010), we suggest that speaker and addressee *jointly* establish which referents are psychologically proximal. Arguably, during interaction interlocutors keep track of the psychological proximity of possible referents. Many contextual factors may contribute to a referent's degree of psychological proximity. For instance, in face-to-face conversations, entities inside the shared space between interlocutors may be experienced as psychologically more proximal than entities outside the shared space (Jungbluth, 2003; Peeters et al., 2015b). An increase in visibility, familiarity, and ownership of possible referents may increase their psychological proximity (cf. Jarbou, 2010; Coventry et al., 2014). Physical and social boundaries between speaker, addressee, and referent may decrease a referent's psychological proximity (Enfield, 2003). Experimental manipulations, informed by careful analysis of everyday demonstrative use, are needed to disentangle the respective contributions of these different contextual influences to the perceived psychological proximity of a referent and the subsequent choice to use one demonstrative and not another.

Furthermore, speakers often organize their use of a demonstrative in relation to their manual pointing behavior (Bangerter, 2004; Cooperrider, in press). Considering demonstrative reference a social undertaking goes hand in hand with its multimodal characteristics. Research on pointing gestures suggests that pointing is often a highly social and communicative act. It has been found that speakers tailor the kinematics of their pointing gesture to the communicative needs of their addressee, for instance by slowing down the stroke and prolonging the hold phase of their gesture for its recognition (Peeters et al., 2015a). Moreover, already in very early stages of life, pointing gestures are often produced with a declarative motive, i.e., to simply share interest in a certain referent and for the addressee to recognize one's communicative intentions (Tomasello et al., 2007). It is hard to unite such a view of pointing as deeply social and communicative with an egocentric view of demonstrative reference in which the speaker is egocentric when choosing a demonstrative. Rather, the social and communicative nature of human pointing confirms that multimodal demonstrative reference is an interpersonal, collaborative process in which the addressee plays a pivotal role.

## Conclusion

Both observational and experimental findings on the production and comprehension of spatial demonstratives suggest that it is now time to move away from an egocentric perspective on spatial demonstrative reference. Demonstratives are better understood in an empirically supported social and multimodal account that considers demonstrative reference a joint action. Such an account fits well within the broader context of referring as a social, interactive phenomenon (Clark and Bangerter, 2004), and is in line with studies looking at joint actions beyond language (e.g., Vesper and Richardson, 2014). A social and multimodal approach to demonstrative reference may also offer new ways to understand how pragmatic language use is acquired in development (Küntay and Özyürek, 2006) and impaired in populations that have difficulties in social interaction and communication.

## Author contributions

All authors listed have made substantial, direct and intellectual contribution to the work, and approved it for publication.

### Conflict of interest statement

The authors declare that the research was conducted in the absence of any commercial or financial relationships that could be construed as a potential conflict of interest.
